# EHMTI-0103. Errors in recognition and management are still frequent in cluster headache

**DOI:** 10.1186/1129-2377-15-S1-C50

**Published:** 2014-09-18

**Authors:** M Sánchez del Río, R Leira, P Pozo-Rosich, JM Laínez, R Alvarez, J Pascual

**Affiliations:** 1 Neurology, Hospital Ruber Internacional, Madrid, Spain; 2 Neurology, Hospital Universitario, Santiago de Compostela, Spain; 3 Neurology, Hospital Universitario Vall de Hebron, Barcelona, Spain; 4 Neurology, Hospital Clínico Universitario, Valencia, Spain; 5 Neurology, Hospital Universitario Central de Asturias, Oviedo, Spain; 6 Neurology, Hospital Universitario Central de Asturias and Ineuropa, Oviedo, Spain

## Introduction

The prevalence of cluster headache (CH) is estimated to be around 0.1% of the general population; that is most likely physicians will be consulted by patients with CH during their clinical practice.

## Aims

To analyse the trajectory to diagnosis and information provided in a series of CH patients from five headache clinics in Spain.

## Methods

CH patients were asked to fulfil an ad hoc questionnaire.

## Results

Seventy-five patients (mean age 41.5 years, 67 males) completed the questionnaire. Patients had visited during an average of 4.9 years a mean of 4.6 physicians who had obtained 2.5 neuroimaging procedures per patient before getting a diagnosis of CH. Sixty-three (84%) had received no diagnosis (21 cases; 28%), while 43 (57%) had been given an average of 2.1 alternative diagnoses. Migraine, trigeminal neuralgia and sinusitis were the most frequent mistakes. After diagnosis, 55% had subjectively received poor/very poor information on CH. Ninety-five percent had poor or incorrect information about the nature of the disease, or acute (70%) and preventive (61%) treatments. Aetiology (90%), management options (36%) and potential adverse events of medications (29%) were their main information demands.

## Conclusions

Although CH is an invalidating and clinically clear-cut disorder suffered by around 1/1000 people, it is still frequently unrecognized and/or mistaken for other disorders, which calls for a better knowledge and education in the diagnosis of the main primary headaches.

No conflict of interest.

